# Iowa

**Published:** 1896-05

**Authors:** 


*Iowa.* The efforts to establish a complete sanitary police for Iowa failed in the recent legislature. The committee having the matter in charge does not feel discouraged, but is already planning for a more active campaign in this direction next session, and with its support better organized it hopes to win the next battle.

